# Can AI-driven games enhance social skills for autistic children? — A three-level meta-analysis

**DOI:** 10.3389/fpsyg.2026.1847426

**Published:** 2026-07-03

**Authors:** Hang Lu, Jiayao Zhao, Yi Zhang, Wei Cheng

**Affiliations:** 1 School of Education, Zhejiang Normal University, Jinhua, China; 2 College of Education for the Future, Beijing Normal University, Zhuhai, China; 3 School of Educational Science and Technology, Nanjing University of Posts and Telecommunications, Nanjing, China

**Keywords:** artificial intelligence, autism spectrum disorder, gamification, social skil, three- level meta-analysis

## Abstract

**Background:**

Gamified interventions for autistic children are becoming increasingly diverse, enhancing their presence, immersion, and sense of accomplishment to improve various social skill indicators. Existing systematic reviews have primarily evaluated intervention effectiveness from the perspectives of game design and intervention objectives. However, these findings are inconsistent and fail to inspect specific improvements in social skills among autistic children.

**Methods:**

To address this gap, this study adopts a three-level meta-analysis approach from the perspective of AI-driven games, examining 14 relevant publications across 9 databases up to March 2026, totaling 37 effect sizes. At the same time, to understand the causes of heterogeneity, the study systematically examined the impact of seven moderating factors on intervention outcomes for autistic children: study design, sample size, intervention goals, measurement tools, intervention frequency, intervention duration, and artificial intelligence technology.

**Results:**

The overall effect size of AI-driven gamified interventions on the social skills of autistic children was *Hedges’g* = 0.511 (95% CI [0.346, 0.677], *p <* 0.001), indicating a moderate positive effect. Specifically, regarding the improvement of social skills in autistic children through AI-driven games, the intervention targeting emotional expression and recognition achieved a moderate and significant effect size (*Hedges’g* = 0.536, *p* < 0.01), while the intervention targeting the dual development of attention and social skills reached a large effect size (*Hedges’g* = 0.963). The remaining skills, such as attention, communication skills, and daily living skills all reached a moderate effect size level (*Hedges’g* = 0.644, 0.650, 0.618). The moderating effect of AI technology was significant (Q(5) = 7.481, *p* < 0.001) and was primarily driven by differences in specific technology types rather than by complex hierarchical structures. Additionally, the study systematically identified 14 gamification elements and distilled the core components, including: feedback mechanisms, reward systems, and level design.

**Conclusion:**

This study provides preliminary evidence that current AI-based gamified interventions can effectively improve the social skills of autistic children. However, it should be noted that this study has certain limitations. Future research should include more high-quality RCTs to explore the validity of intervention effects and systematically examine the optimal configuration of different combinations of AI technologies and gamification elements.

## 1 Introduction

As of 2021, the global prevalence of mental disorders among adolescents aged 10–24 reached 25.47% of the total population, with an incidence rate of 24.35%; Among these, autism ranked eleventh in the years of life adjusted for disability (YLDs) metric (Wang et al., 2025c). Autism spectrum disorder (ASD) is characterized by impairments in social interaction (Luongo et al., 2022), specifically manifesting as limitations in empathy (Soler-Bages, 2025), behavioral imitation, social reciprocity, and higher-order cognition (Gepner and Féron, 2009), leading to repetitive and restricted behaviors (Valente, 2004). This creates a dual challenge in both social development and educational settings. Therefore, maintaining autonomy and independence in educational, occupational, and other life contexts remains a significant challenge for autistic children (López-Bouzas et al., 2024). Although current therapies for autism remain inconclusive (Wang et al., 2025b) and the condition is considered incurable (Luongo et al., 2022). However, technological interventions offer novel strategies and approaches to improve symptoms in autistic children, enhancing their daily living skills, social skills, motor skills (e.g., Lorusso et al., 2025; Priyadharshan et al., 2025), among other areas.

Research shows that approximately 50% of autistic children prefer video games when spending leisure time with peers (Kuo et al., 2013). Accordingly, gamified interventions have been increasingly adopted in autism interventions over the past decade, prompting numerous integrative reviews and meta-analyses. Existing reviews have examined the effects of gamified, serious, and AR/VR-based games on a range of outcomes, including social communication, social cognition, executive functions, emotional regulation, and adaptive skills in autistic children (e.g., Almurashi et al., 2022; Derks et al., 2022; Mittal et al., 2024; Wang et al., 2025b). However, the intervention goals across these studies are not uniform. Given the substantial heterogeneity among autistic children and the varying levels of support afforded by AI technologies (Zhao and Fan, 2025), AI-driven games are often designed to address specific intervention objectives.

Artificial intelligence technologies are revolutionizing the diagnosis and intervention models for autism (Ganggayah et al., 2025), through continuous learning, adaptation, and optimization, they enhance the precision and efficiency of personalized treatment for autistic people (Pandya et al., 2024). However, existing research indicates that current AI-assisted intervention technologies for autistic people still exhibit significant gaps (Muhammad and Rizwan, 2019) and few have undertaken a systematic review from the perspective of AI-driven games. In this study, AI-driven gamified interventions for autistic children are operationalized as: interventions conducted in a gamified format that target core areas of deficit in autistic children, such as social skills, emotion recognition, executive function, and facial recognition; leveraging AI/intelligent sensing technologies such as machine learning (Kinkel et al., 2022), computer vision, natural language processing, intelligent sensing (Mir and Khosla, 2018), adaptive algorithms, and neurofeedback (Xing et al., 2026); and an intervention program in which outcome measures can be quantified and reported via standardized scales or behavioral observations, and for which effect sizes can be calculated.

Currently, AI-driven games have been applied in empirical studies on interventions for autistic children. Consequently, there is an urgent need to systematically review research in this specialized field, comprehensively evaluate the intervention efficacy of AI-driven games, and provide reasonable application recommendations for future AI-driven game design. Based on this, the primary research questions explored in this study are as follows:

- RQ1: What are the main gamification elements in AI-driven games? What are their characteristics?
- RQ2: How effective is the intervention effect of AI-driven gamified interventions on the overall and specific intervention goals of autistic children?
- RQ3: In AI-driven game interventions, how do various moderating variables (such as sample size, intervention conditions, study design, intervention objectives, measurement tools, and AI technology) moderate the AI-driven gamified interventions effect on Social skills for autistic children?

## 2 Literature review

### 2.1 Conceptual framework of AI-driven interventions

With the widespread adoption of the internet, artificial intelligence technologies have accelerated their penetration into everyday life scenarios (Barmpakas and Xinogalos, 2023), gradually expanding into multidisciplinary fields such as medical treatment, sports, and mental health (e.g., Annunziata et al., 2024; Casu et al., 2024; Sierra et al., 2022), thereby enabling numerous intervention activities. Within the context of autism education, AI-driven intervention refers to the use of artificial intelligence technologies to provide tailored, interactive experiences for autistic people (Li et al., 2024). The application of AI technology in the diagnosis and treatment of autistic people represents the most prominent frontier direction. Through capabilities such as precise assessment, real-time adaptation, machine learning, and interface customization, AI can provide personalized assistive technologies for autistic people, significantly enhancing their learning outcomes, communication effectiveness, and overall well-being (Iannone and Giansanti, 2024). Machine learning algorithms distinguish autistic people from children with other developmental disorders, enabling tailored treatment plans. AI-driven augmentation systems, robot-assisted therapy, and serious games enhance autistic people’ developmental potential in social communication, emotional skills, motor skills, and daily living skills (Wankhede et al., 2024), providing robust evidence for subsequent ASD interventions. In recent years, artificial intelligence has expanded its application into the realm of video games due to its multiple advantages in visual enhancement, immersive environment creation, and learning experience optimization (Wu et al., 2023). Related research has also clearly indicated that AI-based serious games hold promising prospects in the healthcare field, capable of accurately assessing user behavioral performance and assisting in disease detection (Abd-alrazaq et al., 2022).

### 2.2 Empirical research on AI-driven interventions

AI technology is progressively empowering diagnosis and intervention for special populations. Empirical studies have leveraged this technology to achieve precise assessment and personalized treatment for these groups. For instance, Ferrer et al. (2024) proposed an AI virtual companion system trained on real-world datasets. Through recorded dialogue practices between adolescents and the AI, Effectively helping autistic individuals recognize cyberbullying. Faria and da Silva Ayrosa (2025) developed a multimodal serious game based on an adaptive neuro-affective system. It aims to capture real-time data from neurodiverse children, including EEG signals and gaming performance, to enhance game engagement. Pandya et al.,'s (2024) research employs POV glasses embedded with computer vision technology, worn by evaluators to non-invasively collect behavioral data during natural interactions, thereby aiding diagnostic assessments.

Multiple systematic reviews and clinical trials have confirmed that autistic people exhibit affinity for technology and interest in games. Consequently, games designed to embed AI technology, construct real-time interactive virtual environments, and incorporate gamified mechanisms to train specific skills through entertainment have become a frontier focus in behavioral intervention research for autistic people (Vacca et al., 2023). For instance, Martins et al. (2026) developed a serious game incorporating machine learning that seamlessly integrates into conventional teaching environments in a non-intrusive, engaging manner. This approach successfully captured behavioral performance in executive function among 3-6-year-old autistic children, enabling timely intervention. Shohieb et al. (2022) developed Pickstar, a mobile educational game based on Dynamic Difficulty Adjustment (DDA) and Adaptive MiniMax algorithms, which effectively enhances vocabulary learning efficiency and duration for autistic children while reducing educators’ assessment burdens. Fan et al. (2025) integrated AI affective computing into the EmoLand interactive game system and developed an iterative design process to guide autistic children in recognizing and expressing facial expressions during social interactions. Consequently, AI-driven games demonstrate a positive trend in application and implementation for autistic children, leveraging advanced technological frameworks to deepen practical applications and expand existing theoretical research.

However, while these studies exhibit distinct technical implementations, the core AI methodologies employed vary significantly. The maturity levels and implementation logics of these technologies differ markedly, making direct comparative evaluations challenging. Furthermore, these interventions focus on highly fragmented skill dimensions, lacking a unified assessment framework and standardized measurement criteria. Meta-analysis, as a powerful statistical method, enables the quantitative synthesis of effect sizes from multiple intervention or treatment studies, yielding more reliable and comprehensive conclusions (Shim et al., 2019). Through meta-analysis, researchers can integrate data on the skill-enhancing effects of AI-driven gamified interventions across different studies for autistic children. This approach clarifies the relative strengths and limitations of various AI technologies, thereby providing more precise design guidance and optimization directions for future AI-driven gamified interventions.

### 2.3 Moderating variables influencing AI-driven gamified interventions

Prior to conducting a meta-analysis, researchers must systematically code all aspects of included studies as potential moderating variables for incorporation into statistical models, following the perspective of Pigott and Polanin (2020). Given the inherent complexity of AI technology, gamified interventions inevitably involve multiple moderating variables during implementation. Simultaneously, interventions targeting autistic people constitute complex systems where effectiveness is shaped by the interplay of factors including intervention measures and characteristics, intervention goals, intervention quality, duration, and frequency (e.g., Schreibelmayr and Mara, 2025; Silva et al., 2025; Yang et al., 2025; Zhou et al., 2025). Specifically, intervention outcomes may vary significantly based on Intervention Duration and participant age (Wang et al., 2026). Additionally, study design serves as a crucial moderating variable. Duan et al. (2025) noted that randomized controlled trials (RCTs) consistently reported small to moderate effects of digital interventions in improving sleep quality among autistic individuals, compared to non-randomized controlled experiments. Further reviews identify measurement tools and sample size as critical variables influencing outcomes (Yang et al., 2025), while Ji et al.,'s (2025) scoping review also incorporates information on study design, participant numbers, and intervention measures. Therefore, to systematically evaluate the impact of AI-driven gamified interventions on autistic children, a holistic approach is essential. This requires explicitly identifying all potential moderating variables to ensure the integrity and interpretability of moderation analyses.

### 2.4 Present study

Current reviews and meta-analyses of gamified interventions for autistic people have evaluated and categorized their effectiveness across three levels of technological delivery: traditional games, computer games, and VR/AR immersive games. Regarding traditional games, while not reliant on high-tech equipment, they can stimulate emotional responses and improve interpersonal relationships by providing intuitive social interactions and contextual experiences (Fang et al., 2016). A meta-analysis of 19 randomized controlled trials on gamification revealed that game-based interventions significantly enhance the play skills of autistic children (*g* = 0.439) (Kent et al., 2020). Related review literature also indicates that non-technical collaborative activities like hide-and-seek and LEGO play effectively enhance social and collaborative abilities in autistic children (Khatab et al., 2024). Regarding computer games, multiple review studies confirm their positive effects on improving attention, reasoning and memory abilities, social skills, and motor skills in autistic children (e.g., Almurashi et al., 2022; Carneiro et al., 2024; Tsikinas and Xinogalos, 2019). Additionally, concerning VR/AR gamified interventions. a meta-analysis indicates that VR-based interventions significantly improve autistic individuals’ overall abilities, with particularly pronounced effects on daily living skills (*g* = 1.15) (Karami et al., 2021). Multiple reviews further highlight the clear potential of such immersive games in enhancing autistic individuals’ cognitive, social, and learning skills (e.g., Mittal et al., 2024; Toma et al., 2024). Thus, all three technological approaches positively influence functional improvement in autistic people. Building upon this foundation, to further explore emerging technological pathways in gamified interventions, it is necessary to systematically examine the efficacy of AI-driven gamified interventions to assess the potential value of AI technology in terms of intervention effectiveness and rationality.

## 3 Methods

### 3.1 Literature search and inclusion strategy

This study searched nine databases: Scopus, PubMed, ScienceDirect, Web of Science, IEEE Explore, Springer Nature Link, Engineering Village, ERIC, and Google Scholar. Following the PICOS framework (Table 1) and using the search term “(“Autism Spectrum Disorder” OR ASD OR autism OR autistic disorder OR “autistic traits”)AND(child OR pediatric)AND(“artificial intelligence” OR AI OR “machine learning” OR “computer vision” OR “deep learning” OR “neural network” OR “adaptive learning” OR “affective computing” OR “intelligent tutoring” OR “AI-driven”)AND(game OR gamification OR “serious game” OR “educational game”).” Due to the limited number of available studies, the search time frame was set from the inception of each database to March 2026. A total of 1,326 articles were identified.

**Table 1:** PICOS retrieval principles.

Element	Content
P (Population)	Autistic Children
I (Intervention)	AI-driven gamified intervention (including machine learning, computer vision, etc.)
C (Comparison)	Control groups (conventional intervention, no intervention, pre-intervention baseline, etc.)
O (Outcome)	Social skills, attention, emotional recognition and expression, communication abilities, and interactive capabilities
S (Study)	Empirical research

### 3.2 Literature screening process

**Figure 1:**
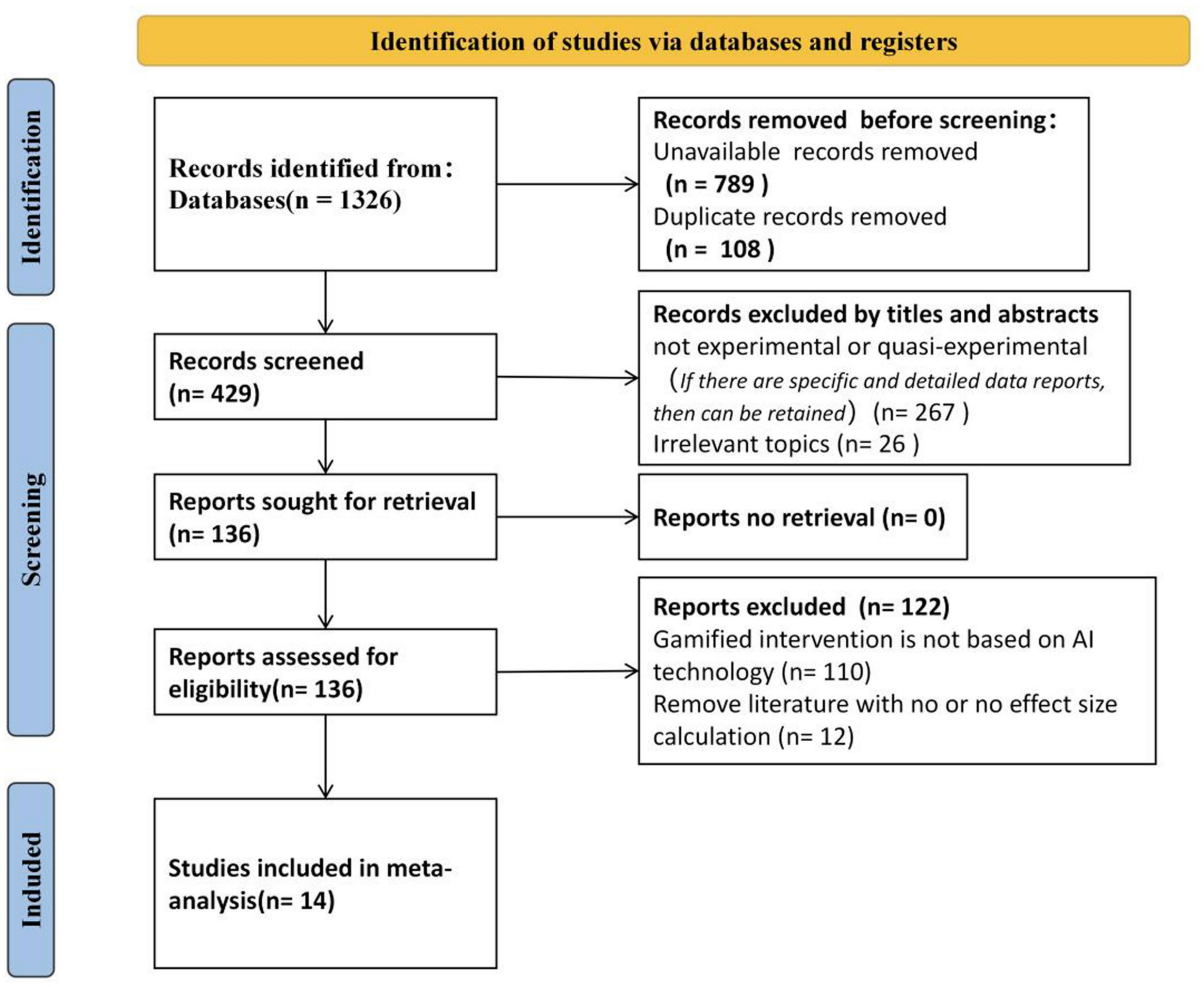
PRISMA Flowchart of the Screening Process.

The study selection followed the PRISMA 2020 framework, as illustrated in Figure 1. The 1,326 retrieved articles underwent preliminary screening based on the following criteria: 789 inaccessible articles and 108 duplicate articles were excluded based on their titles. The remaining articles were further processed as follows: (1) After reviewing titles and abstracts, 267 non-experimental or quasi-experimental studies (if there are specific and detailed data reports, then can be retained) and 26 irrelevant studies were excluded. (2) Following full-text review, 110 studies lacking AI-based gamification interventions were discarded. Among the remaining 26, studies without calculable effect sizes were excluded, yielding 14 final articles.

### 3.3 Literature extraction and coding

This study further extracted thematic information from the 14 included articles, including research descriptions, study subjects, intervention duration, research design, gamification elements, intervention objectives, and AI technologies. To achieve more intuitive presentation, specific codes were assigned to each element, ensuring each possesses a unique identifier. The detailed coding scheme is presented in Table 2. This process yielded the foundational information for all 14 articles.

**Table 2:** Literature coding scheme table.

Theme	Extract elements	
Research description item	First author of the literature, year	
Research subject	Sample size (0–50 people, 50–100 people, ≥100 people)	
Intervention duration	Intervention frequency (high-frequency, low-frequency, medium-frequency), Intervention Duration (≤3 weeks, 3–6 weeks, 6–9 weeks, 9–12 weeks, 12–18 weeks)	
Study design	Non-Randomized Controlled Trial (NRCT), Randomized Controlled Trial (RCT), Single-Group Pre-Post Design (PREPOST)	
Gamification elements	Primary indicators	Secondary indicators
	Dynamic elements	Personalization (EP), Rewards (ER), Collaboration (EO), Interaction (EN), Robot (EH)
	Contextual elements	Real-life Situations (EL) Narrative (ET)
	Construct elements	Feedback (EF), Monitoring (ES), Imitation (EI), Level Design (EC), Challenge (EB), Dynamic Adjustment (ED)
	Other	Parent Involvement (EA)
Intervention goals	Emotion regulation and emotional disorders	Emotion Recognition and Expression (DE), Anxiety (DN)
	Social interaction and communication	Social Skills (DS), Communication Skills (DC), Interaction (DI), Daily living skills (DD)
	Computational science	Attention (DA)
Measurement tool categories	Survey Questionnaire (SURV), Scale (SCALE), Behavioral Observation (OBSERVE), Hardware Device (DEVICE), Behavioral Performance (BEHAVE), test (TEST)	
AI technology	Machine Learning (TM), Dynamic Adjustment (TD), Deep Learning (TL), Neural Feedback (TS), Adaptive Learning Technology (TA), Computer Vision (TV)	

In this study, the intensity of game therapy was stratified based on the weekly frequency of interventions: high-frequency indicated game interventions conducted every day of the week; medium-frequency indicated at least three game interventions per week; and low-frequency indicated fewer than three interventions per week. Intervention duration was defined according to the plan outlined in UCB’s 2022 Statistical Analysis Plan (Study PA0010) published on ClinicalTrials.gov. Five intervention duration categories were included in this study: ≤3 weeks, 3–6 weeks, 6–9 weeks, 9–12 weeks, and 12–18 weeks. The classification of gamification elements referenced the four-category framework proposed by Wang et al. (2025b), with expansions made to secondary indicator elements. For intervention goal categorization, three categories were selected from Karami et al. (2021) classification of social and communication skills, emotion recognition and regulation skills, daily living, and computer science. Research design, AI technology, and measurement tools were directly extracted from the literature and subsequently systematically coded.

### 3.4 Quality assessment

**Figure 2:**
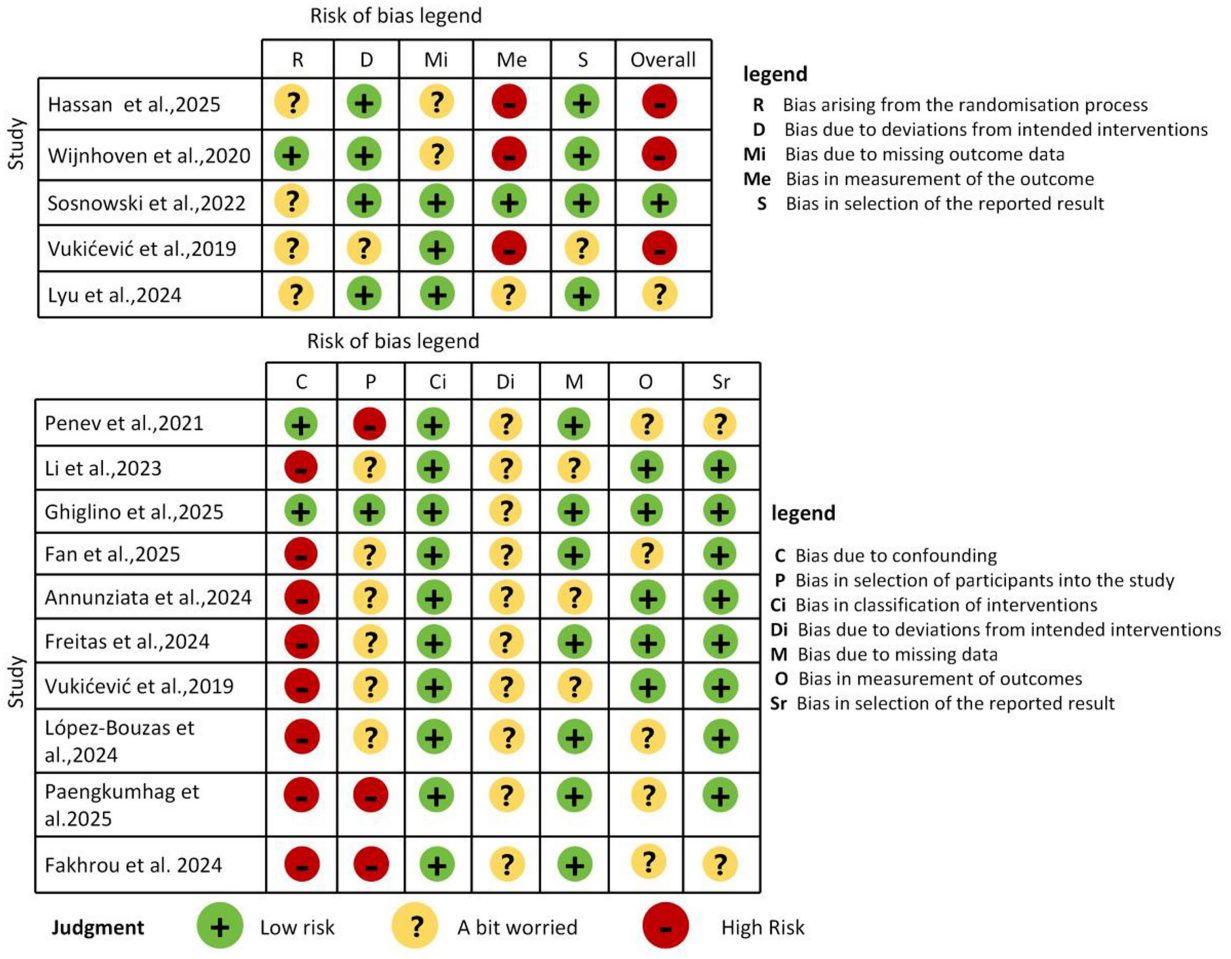
Traffic light diagram for risk of bias assessment.

Given that this study comprehensively included randomized and non-randomized controlled trials as well as single-group pre-post studies, a stratified assessment strategy based on study design type was adopted to ensure the rigor and appropriateness of literature quality evaluation. For randomized controlled trial literature, assessments strictly followed the Cochrane RoB 2 tool updated and released by Sterne et al. (2019). This tool first defines the intervention outcomes to be assessed and organizes all information sources. It then proceeds to evaluate each item systematically based on predefined questions across five core domains, assigning a risk of bias judgment of “low/high risk, some concern.” For non-randomized controlled trials and single-group pre-post studies, the ROBINS-I assessment framework (Sterne et al., 2016) is applied to conduct a structured evaluation across seven potential bias domains. Furthermore, for results encompassing multiple study designs within the research by Vukićević et al. (2019), quality assessment strictly applies the corresponding evaluation tools to each specific study design associated with each effect size. Literature quality assessments were conducted independently by two researchers. Disagreements were resolved through third-party mediation until 100% consensus was reached. The final assessment results are presented in Figure 2.

Based on the overall information presented in the figure, the included studies exhibit heterogeneity in quality. The subsequent meta-analysis will fully consider these bias risk assessments and explore the potential impact of studies of varying quality on the pooled effect size through sensitivity analyses.

### 3.5 Data analysis

This study included 14 publications, yielding a total of 37 effect sizes, indicating multiple effect size measures within the same study. Due to factors such as measurement tools, the interdependence among effect sizes cannot be ignored. Traditional meta-analysis tends to overestimate the standard errors and heterogeneity of the measured effect sizes. Given the diversity in specific AI implementation methods and intervention objectives across the included studies, this approach risks facing criticism that effects cannot be attributed to the tools themselves, leading to difficult-to-interpret results (Weidlich et al., 2025). However, this study does not stop at calculating the overall effect size. Instead, it further analyzes effects using moderating variables such as “AI technology” and “intervention purpose.” This analysis examines the extent to which the heterogeneity in overall effect sizes is attributable to moderating variables, going beyond simple pooling to clarify the contributions of different moderators to the overall effect.

In addition, a three-level meta-analysis more appropriately handles nested data structures. It simultaneously captures sampling error (Level 1), dependencies among effect sizes within the same study (Level 2), and inter-study heterogeneity (Level 3), thereby providing more robust estimates (Cheung, 2014). Therefore, this study evaluated the included literature using the three-level meta-analysis tutorial by Assink and Wibbelink (2016). Since the included studies did not directly provide effect size data and varied in research design and total sample size, this study first used CMA version 3.7 software to calculate and convert effect sizes into the corrected *Hedges’g*. Subsequent specific three-level meta-analysis procedures were then conducted in the RStudio visualization and programming software.

Heterogeneity tests assess the consistency of effect sizes across meta-analysis studies, typically quantified by two metrics: the absolute measure—*τ*^2^—directly reflecting effect size variability; and the relative measure—*I*^2^—indicating the proportion of total variation attributable to heterogeneity (Nakagawa et al., 2023). *I*^2^ is categorized as follows: 0–25% indicates low heterogeneity, 25–75% indicates moderate heterogeneity, and 75–100% indicates very high heterogeneity (Higgins et al., 2003). However, if *I*^2^ approaches thresholds or is accompanied by wide confidence intervals, it should be interpreted in conjunction with the Q-statistic and corresponding *p*-value. Publication bias also undermines meta-analysis validity, necessitating its assessment (Rothstein et al., 2005). To enhance meta-analysis reliability, researchers commonly employ funnel plots, loss-to-follow-up coefficients, Egger’s test, and trimming methods to assess publication bias (Zuo et al., 2025). This study also selected some of these methods for corresponding data processing.

## 4 Results

### 4.1 Description of included studies

This study included a total of 14 independent studies (Penev et al., 2021; Li et al., 2023; Hassan et al., 2025; Ghiglino et al., 2025; Fan et al., 2025; Annunziata et al., 2024; Freitas et al., 2024; Wijnhoven et al., 2020; López-Bouzas et al., 2024; Sosnowski et al., 2022; Vukićević et al., 2019; Lyu et al., 2024; Paengkumhag et al., 2025; Fakhrou et al., 2024), spanning the years 2019 to 2025 and covering recent research advances in the application of AI technology in the field of skills intervention. In terms of study design, the included studies employed various types, including randomized controlled trials (RCTs), non-randomized controlled trials (NRCTs), and pre-post studies (PREPOST). Among these, RCTs exhibit the highest methodological rigor; NRCTs are often used for preliminary exploratory assessments of intervention effects and, although lacking randomization, can provide initial indications of effectiveness; pre-post studies evaluate intervention effects by comparing pre- and post-intervention measurements, but they are limited by the absence of a control group to account for confounding factors.

**Figure 3:**
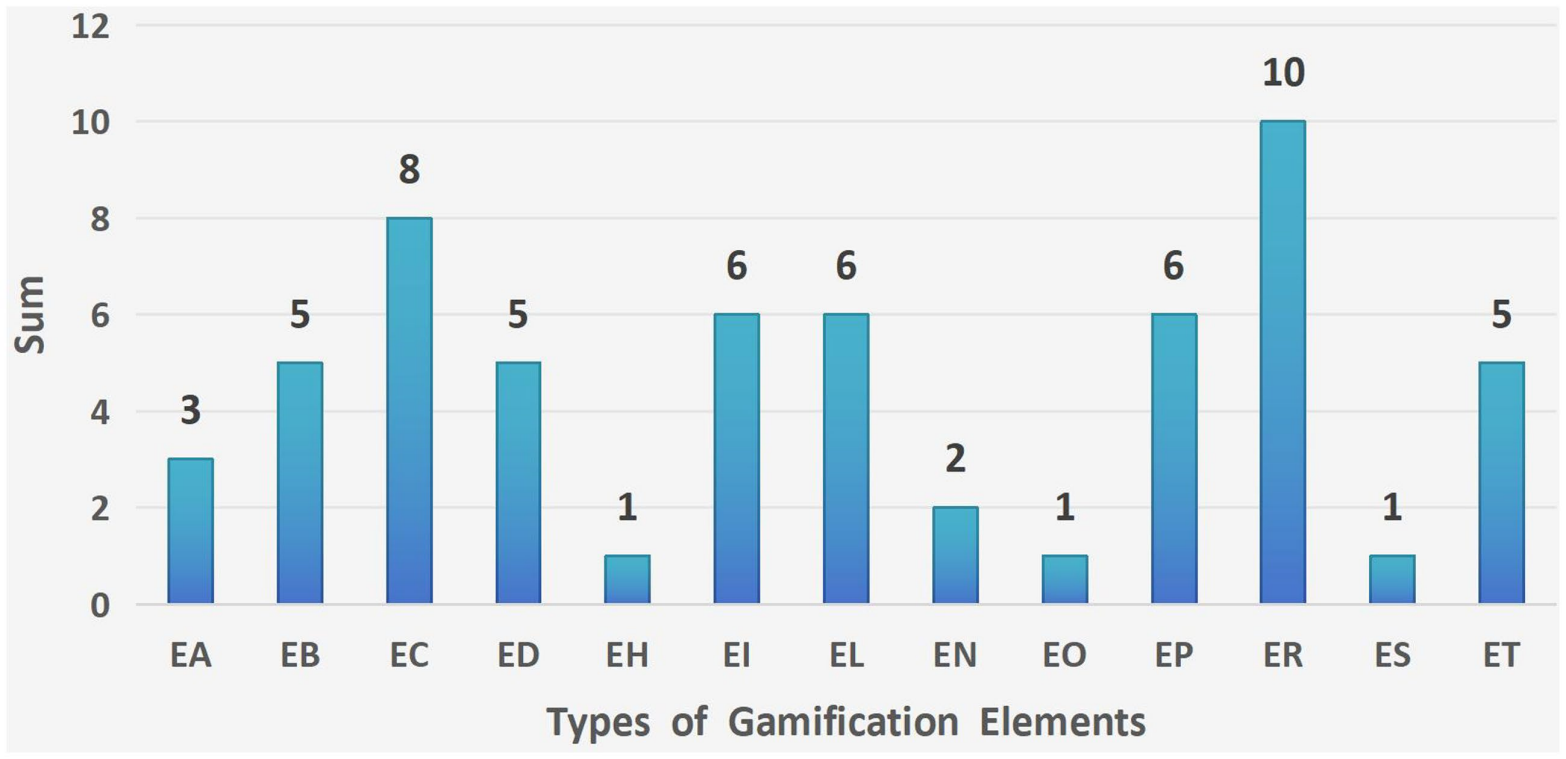
Statistics of gamification elements. EF, feedback; ER, reward; EB, challenge; ED, dynamic adjustment; EL, real-life scenario; ET, narrative; EO, cooperation; EN, interaction; EH, robot; EP, personalization; ES, monitoring; EI, imitation; EC, level design; EA, parental involvement.

Regarding gamification elements, Wang et al. (2025b) categorized game elements into ten components, including feedback, rewards, personalized learning, monitoring, and personalization, and provided detailed explanations for each. Building upon these elements, this study expanded and supplemented them to form a comprehensive gamification model comprising fourteen specific components (Figure 3), while also quantifying the frequency of occurrence for each gamification element and its corresponding description (Table 3). In the statistical analysis, gamification elements with an occurrence frequency exceeding 50% primarily included feedback, rewards, and levels. These high-frequency elements show significant overlap with the findings of Wang et al., indicating consistency in the composition of core gamification elements. This suggests that these fourteen specific elements broadly cover current gamification elements and possess a certain degree of validity.

**Table 3:** Description of gamification elements and frequency statistics.

Element	Frequency	Description
Feedback	85.71%	Provides goal progress (e.g., text, audio cues) to enhance participants’ awareness of their actions
Reward	71.43%	Reinforce positive behaviors with points, virtual items, etc., to boost participation willingness
Challenge	35.71%	Set appropriately challenging goals or levels
Dynamic adjustment	35.71%	Managers can adjust game difficulty or content in real time based on player performance
Real-life scenarios	42.86%	Integrating game tasks with real-life scenarios
Narrative	35.71%	Providing emotionally driven context through storylines, character development, and plot progression
Level design	57.14%	Gradually escalating difficulty to match participant skill levels
Collaboration	7.14%	Foster peer interaction (e.g., multiplayer games)
Interaction	14.29%	Offering multiple interaction methods
Robot	7.14%	Guides, prompts, or accompanies players using virtual characters or AI assistants
Personalization	42.86%	Customizing tasks/difficulty based on autistic individuals’ differences
Monitoring	7.14%	Tracking intervention progress via parent/teacher devices
Imitation	42.86%	Players observe and replicate observed expressions, behaviors, etc.
Parent involvement	21.43%	Design features allowing parents to view, evaluate, or co-participate

### 4.2 Publication bias assessment

**Figure 4:**
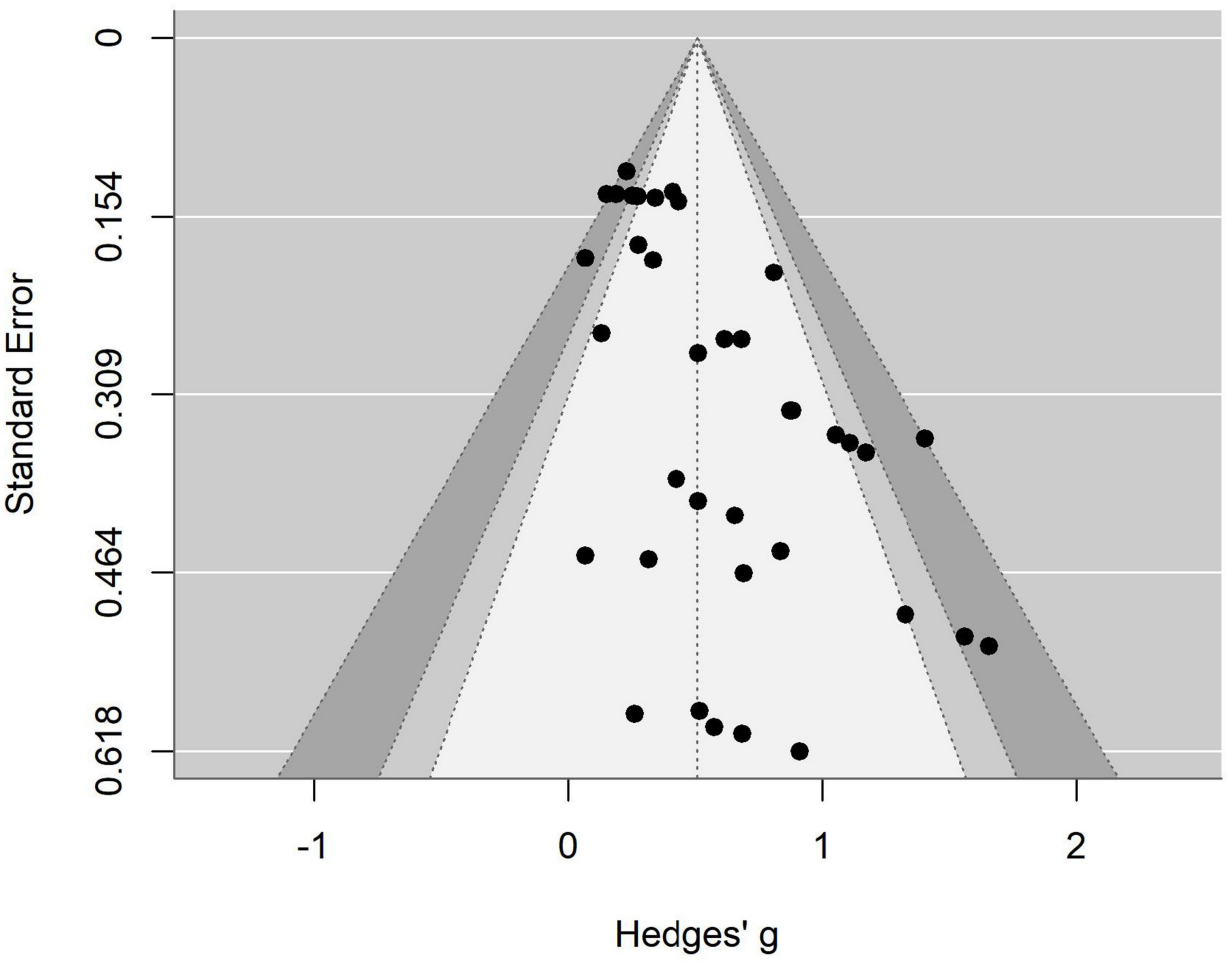
Publication bias funnel plot.

To assess publication bias, a funnel plot was constructed (Figure 4). The effect sizes in this plot were broadly symmetrically distributed around the overall effect, showing no significant skewness to either side. Furthermore, the calculated margin of safety coefficient *N* = 1927, significantly exceeding the empirical threshold of 5 × K + 10 (K = 37), further supporting the conclusion that no publication bias was present. Based on the combined visual assessment and quantitative analysis of the safety margin coefficient, this study demonstrates no significant publication bias.

### 4.3 Main effect analysis and heterogeneity test

**Figure 5:**
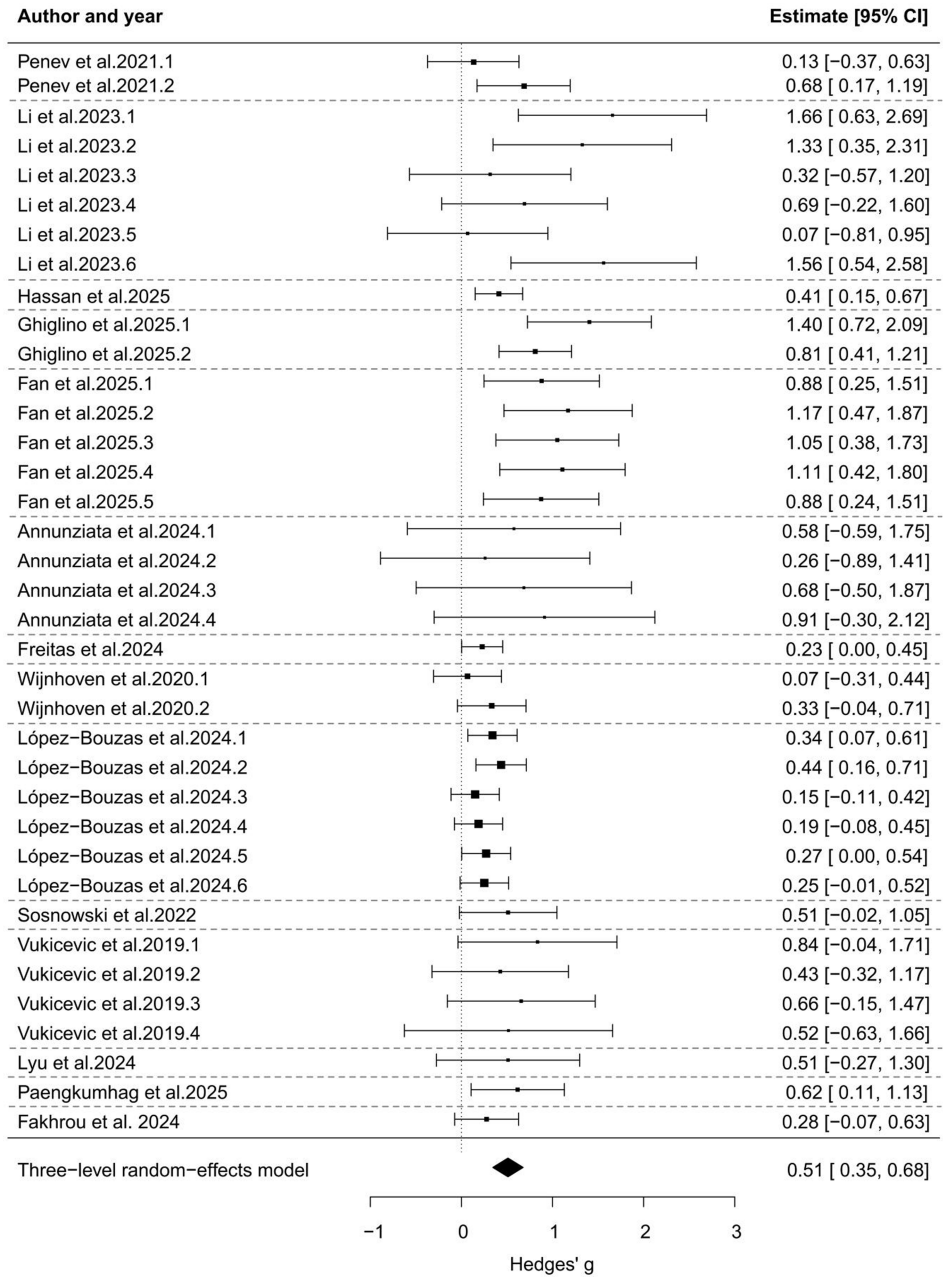
Forest plot of effect sizes and confidence intervals across studies.

The main effect analysis revealed that AI-driven games demonstrated a moderate effect size for autistic children, with *Hedges’g* = 0.511, standard error = 0.082, and a 95% confidence interval (CI) of [0.346, 0.677]. Heterogeneity testing (Table 4) revealed a significant Q-test statistic (*p* < 0.001), indicating substantial overall effect heterogeneity. The hierarchical heterogeneity decomposition yielded the following results: sampling error (level 1) accounted for 0%; within-study variance (level 2) was 0.00 with an *I*^2^ of 0%, indicating no significant heterogeneity; between-study variance (level 3) was 0.057 (*p* < 0.001) with an *I*^2^ of 52.53%. These stratified results indicate that the observed heterogeneity primarily stems from differences between studies, while within-study heterogeneity is negligible. Based on this, further analysis of moderating variables can be conducted. To visually compare effect sizes across studies, a forest plot is presented in Figure 5.

**Table 4:** Results of heterogeneity tests among studies.

Indicator	Heterogeneity				Tau^2^		*I* ^2^	
	*Q*	df(Q)	*p*	*I*^2^(level 1)	level 2	level 3	level 2	level 3
result	64.039	36	0.003	0%	0.00	0.057	0.00%	52.53%

### 4.4 Sensitivity analysis

To further assess the robustness of the meta-analysis results, a sensitivity analysis was conducted. First, we examined the impact of individual studies on the pooled effect size using a stepwise exclusion method; the results showed that no single study led to a fundamental change in the conclusions. Second, to assess the impact of study design types on the results, we excluded pre-post studies (PREPOST) and re-conducted the three-level meta-analysis (Table 5). The pooled effect size estimate changed from 0.511 (95% CI: 0.346–0.6767, *p <* 0.001) to 0.552 (95% CI: 0.310–0.795, *p* = 0.0001). The direction of the effect remained consistent, and statistical significance was unchanged. However, the level of heterogeneity decreased significantly (Q = 64.04 (*p* = 0.003) to 29.31 (*p* = 0.045)), and between-study variance decreased (from 0.057 to 0.036). This result indicates that pre-post study designs are a major source of heterogeneity; however, after excluding them, the main effect remained significant and exhibited a moderate effect size. This suggests that the meta-analysis results are not sensitive to the types of study designs included and that the conclusions are robust.

**Table 5:** Sensitivity analysis excluding pre- and post-test studies.

Indicator	*Hedges’g*	SE	95% CI	*p*	Heterogeneity	*τ*^2^_level3
Main effect analysis	0.511	0.082	[0.346, 0.677]	<0.001	64.04 (df = 36, *p* = 0.003)	0.0574
Sensitivity analysis	0.552	0.115	[0.310, 0.795]	0.0001	29.31 (df = 18, *p* = 0.045)	0.0356

### 4.5 Moderation effect tests

The study further examined the moderating effects of sample size, research design, intervention purpose, intervention frequency, measurement tools, Intervention Duration, and AI technology. Results of the moderation effect analysis (Table 6) indicated that neither study design (Q(2) = 1.293, *p* = 0.288), sample size (Q(2) = 1.796, *p* = 0.181), nor intervention purpose (Q(6) = 1.164, *p* = 0.355), measurement tools (Q(5) = 0.433, *p* = 0.822), intervention frequency (Q(2) = 1.499, *p* = 0.238), and Intervention Duration (Q(3) = 0.398, *p* = 0.755) did not reach statistical significance. Conversely, the moderating effect of AI technology (Q(5) = 7.481, *p* < 0.001) was significant, indicating that AI technology played a crucial moderating role in this study. Since only a single moderator variable showed a significant effect, this study did not examine multicollinearity among moderator variables (Assink and Wibbelink, 2016).

**Table 6:** Results of moderation effect tests.

Moderators	ES	Intercept/*Hedges’ g*	95% CI	*β*	Omnieus test	*p*	Var.1evel2 Var.1evel3
Sample size					Q(2) = 1.796	0.181	0.00 0.048
0–50 people	25	0.631***	0.425, 0.836				
50–100 people	9	0.360	0.039, 0.681	−0.270*			
≥100 people	3	0.306	−0.063, 0.675	−0.325*			
Intervention frequency					Q(2) = 1.499	0.238	0.00 0.048
Low frequency	21	0.413***	0.209, 0.617				
Mid frequency	14	0.692	0.425, 0.959	0.279*			
High frequency	2	0.401	−0.177, 0.979	−0.012			
Intervention duration					Q(3) = 0.398	0.755	0.00 0.076
≤ 3 weeks	18	0.410**	0.140, 0.681				
3–6 weeks	11	0.572	0.252, 0.891	0.161*			
6–9 weeks	4	0.630	0.236, 1.025	0.220*			
12–18 weeks	4	0.600	−0.230, 1.430	0.190			
Research design					Q(2) = 1.293	0.288	0.00 0.058
RCT	6	0.376*	0.059, 0.694				
NRCT	13	0.737	0.392, 1.082	0.361*			
PREPOST	18	0.491	0.268, 0.714	0.115			
Purpose of intervention					Q(6) = 1.164	0.355	0.00 0.120
DE	17	0.536**	0.221, 0.850				
DA	4	0.644	0.122, 1.166	0.108			
DA + DS	2	0.963	0.165, 1.760	0.427*			
DN	2	0.199	−0.566, 0.963	−0.337*			
DI	3	−0.166	−0.910, 0.577	−0.702**			
DC	4	0.650	−0.018, 1.318	0.114			
DD	1	0.618	−0.274, 1.510	0.082			
Measurement tool					Q(5) = 0.433	0.822	0.00 0.061
SCALE	16	0.546***	0.257, 0.836	0.04			
BEHAVE	1	0.513	−0.237, 1.263	−0.033			
DEVICE	2	0.497	−0.002, 0.995	−0.050			
OBSERVE	7	0.758	0.316, 1.200	0.211*			
SURV	9	0.413	0.080, 0.745	−0.134			
TEST	2	0.346	−0.175, 0.868	−0.200*			
AI technology					Q(5) = 7.481	<0.001	0.00 0.00
TV	17	0.295***	0.200, 0.391				
TA	4	0.780**	0.518, 1.042	0.485***			
TM	1	0.412	0.141, 0.683	0.117*			
TS	2	0.199	−0.087, 0.474	−0.097			
TV + TL	11	0.952***	0.707, 1.197	0.657***			
TM + TV	2	0.316	−0.017, 0.649	0.021			

ES, effect size; CI, confidence interval; β, estimated regression coefficient; Var.level 2, within-study variance; Var.level3, between-study variance; ^*^p < 0.05; ^**^p < 0.01; ^***^p < 0.001; NRCT, non-randomized controlled trial; RCT, randomized controlled trial; PREPOST, pre-post single-group design; DE, emotion recognition and expression; DN, anxiety; DS, social skills; DC: communication skills; DA, attention; TC, convolutional neural network; TM, machine learning; TD, dynamic tuning; TL, deep learning; TS, neural feedback; TA, adaptive learning technology; TV, computer vision.

## 5 Discussions

### 5.1 Analysis of gamification elements in AI-driven games

#### 5.1.1 Overview of gamification element implementation

Gamification elements can enhance children’s online behavioral engagement and learning performance through task appeal (Huber et al., 2023); However, the boundaries of cognitive resources among autistic children are not uniform (Mayer, 2005). Different game design elements may either reinforce or cancel each other out in their effects, leading to mixed outcomes for gamification projects (Mazarakis and Bräuer, 2023). Therefore, the perspective on gamification elements should shift from holistic to granular analysis, with clear delineation of distinct gamification components.

This study conducted frequency analysis of gamification elements identified across the 11 extracted articles. Among these, feedback (85.71%), rewards (71.43%), and Level Design (57.14%) accounted for the highest proportions (≥50%). This indicates that games in the 11 studies frequently employ level-based progression, provide real-time feedback on children’s progress, and use rewards to encourage autistic children’s participation, aiming to achieve the intended intervention outcomes. Real-life scenarios, imitation, personalization accounted for 42.86%, while narrative, challenge, and dynamic adjustment each accounted for 35.71%. This demonstrates that AI-based gamified interventions for autistic children promote skill transfer by simulating real-life situations and utilizing modeling and imitation. At the same time, role-playing and narrative cues can stimulate emotional engagement and sustained participation in autistic children; challenge design and dynamic adjustments ensure that the game’s difficulty remains within the child’s “zone of proximal development,” thereby achieving personalized adaptation. Furthermore, relevant meta-analyses indicate that subgroup effects are higher in natural environments compared to laboratory settings (Tupou, 2020). Embedding interventions in natural environments, along with gamification elements and the integration of AI technology, can consolidate intervention outcomes and enhance their retention. In addition, five gamification elements, collaboration (7.14%), interaction (14.29%), robotics (7.14%), monitoring (7.14%), and parental involvement (21.43%), were used less frequently. This is because they serve as supplementary elements rather than primary gamification components; the underlying rationale is to expose autistic children to authentic interactive experiences, stimulate their interest in the game, and enable them to complete game tasks.

Current gamification interventions have shown certain patterns in the use of elements; high-frequency elements such as feedback, rewards, and level design contribute to some extent to autistic children’s task engagement and sustained motivation. However, existing research mostly focuses on “near-transfer” effects, and there is currently no mature, applicable approach for systematically combining various gamification elements to generate synergistic effects.

#### 5.1.2 Extraction of core game elements and their characteristic descriptions

Among numerous gamification elements, this study identifies those appearing in over 50% of cases—feedback, rewards and levels—as core game elements. The specific characteristics of these three core elements are as follows: Feedback aims to provide educators (teachers/parents) and researchers with information about autistic children’s performance and status (Mazarakis and Bräuer, 2023) to enable timely adjustments to task contexts and level difficulty, adapting to the child’s practice pace and enhancing engagement. Rewards utilize game systems such as points and unlockable skins to express recognition of the child’s current behavioral performance (Hallifax et al., 2020), motivating sustained engagement in specific game behaviors. Levels provide a progressive skill training framework for autistic children through tasks of varying difficulty and content. Examples include training eye contact via role-interaction tasks or enhancing sustained attention through sequencing games, ensuring training content dynamically aligns with children’s developmental levels.

The core gamification elements identified in this study show high consistency with Wang et al.,'s classification, both acknowledging feedback as a key gamification element. This corresponds to the feedback and narrative elements extracted by Govender and Arnedo-Moreno (2021) for digital games. The reward element ranked second among the 14 elements, mirroring its adoption rate of 81% in Hong et al. (2024)'s study of 23 game elements, indicating comparable prominence. The remaining elements also exhibit substantial overlap with the 23 elements extracted by Hong et al., Thus, the gamification elements identified in this study have not only been repeatedly validated in prior research but also demonstrate strong adaptability and effectiveness in practical applications.

### 5.2 Evaluation of the intervention efficacy of AI-driven gamified interventions for autistic children

Using R 4.3.2 software, this study conducted a quantitative meta-analysis of three levels on the findings from 14 original literature sources. Results indicate that AI-driven game interventions yield a moderate overall effect size for autistic children (*g* = 0.511, *p* < 0.001), statistically validating the intervention efficacy of AI-driven games for this population. In addition, this study refines the intervention objectives into seven categories: emotion recognition and expression, anxiety, social skills, communication skills, interaction, daily living skills and attention, and explores the effects of each intervention objective as follows.

An effect size analysis for different intervention targets showed that, with emotional recognition and expression (DE) as the baseline, the effect size was *g* = 0.536 (95% CI: 0.221, 0.850, *p* < 0.01), indicating that AI-driven games produce a moderate and significant effect on this target. Studies targeting the dual development of attention and social skills (DA + DS) showed a large effect size (*g* = 0.963, 95% CI [0.165, 1.760]). For some single intervention targets, moderate effect sizes were observed (*g* = 0.644, 0.650, 0.618), but the 95% confidence intervals mostly included 0 or were close to 0, failing to reach statistical significance. This was the case for studies on attention (DA), communication skills (DC), and daily living skills (DD). This suggests that the effectiveness of AI-driven game interventions for these targets requires further validation. In contrast, the intervention effects for anxiety (DN) and interaction skills (DI) did not reach the desired level, with effect sizes significantly lower than expected.

These hypothesis-generating observations are consistent with the core characteristics of autistic children, which primarily involve deficits in social interaction, communication, and empathy (Wang et al., 2025a); consequently, there has been extensive research focusing on emotion recognition and expression in autistic children, and intervention efforts in this area have yielded significant results. The negative effects observed on the interaction skills dimension suggest that current AI-driven game interventions have significant limitations in helping autistic children generalize skills learned within the game to real-world social settings (O’Keeffe and McNally, 2023). The outcome measure DI in this study assesses interactive abilities in real-world environments, whereas most AI games only train interactive behaviors in virtual or laboratory settings, indicating a significant skill generalization gap between the two. The findings of Jouen et al. (2017) also support this conclusion, as they found that conventional interventions combined with gamified interactive training did not significantly improve real-world interactions or cognitive functions in autistic children. Thus, it is evident that improvements in social interaction skills among autistic children rely more on real-life interpersonal interactions than on virtual game interactions on a screen.

Furthermore, attention serves as a gateway to enhancing the effectiveness of interventions for autistic children (Mehmood et al., 2021), and limitations in attention allocation, sustained focus, or attention shifting can hinder the multidimensional development of autistic children in areas such as language, social skills, and cognition (Todd and Bahrick, 2023). Related research also identifies joint attention as a foundational skill for social interaction development, which, when combined with peer mediation, forms an integrated training context (Hansen et al., 2023). Further in-depth exploration of this area is warranted.

### 5.3 Analysis of moderating effects on AI-driven game intervention outcomes for autistic children

The results of the moderation analysis may be limited by the small number of effect sizes included in the analysis, leading to insufficient statistical power. Furthermore, the 95% confidence intervals for most subgroups overlap, which to some extent weakens the statistical significance of the estimated moderation coefficients. Given these limitations, our conclusions represent only preliminary exploratory evidence and require more systematic evaluations in future studies for further validation.

#### 5.3.1 Research design and sample size

The results of the moderation analysis showed that neither study design nor sample size reached overall significance as moderator variables (*p* > 0.050). However, subgroup analysis indicated that the non-randomized controlled trial subgroup exhibited a moderate-to-large effect size (*Hedges’ g* = 0.737, *p* < 0.05); the small-sample subgroup (*n* = 0–50) showed a more significant effect compared to other sample sizes (*g* = 0.631, *p* < 0.001). These findings can serve as preliminary exploratory results to inform future experimental designs for interventions targeting autistic children. Overall, all subgroups exhibited a positive trend in effect size, but their combined contribution was insufficient to achieve statistical significance for the overall moderation effect.

#### 5.3.2 Intervention frequency and intervention duration

Intervention frequency (*p* = 0.238) and intervention duration (*p* = 0.755), when considered as moderator variables, did not reach statistical significance at the overall level, indicating that these two variables have limited explanatory power regarding the effect size of AI-driven game interventions.

Although the overall moderating effects were not significant, subgroup analyses revealed comparability among intervention frequency variables: the effect sizes and statistical significance of low-frequency and high-frequency interventions (*g* = 0.413, *p* < 0.001; *g* = 0.401, *p* > 0.050) were both significantly lower than those of medium-frequency interventions (*g* = 0.692, *p* < 0.050). This is consistent with the findings of Linstead et al. (2017), who noted a 35% linear relationship between intervention intensity and treatment outcomes for autistic children, with moderate intervention intensity being more advantageous for promoting learning outcomes compared to either too low or too high intensity. Similarly, physical interventions for autistic children have also shown that moderate-to-high-frequency interventions yield the best results (Yang and Li, 2025). However, this contradicts the conclusion reached by Kouroupa et al. (2022), who found that intervention frequency does not modulate outcomes for autistic children.

All four duration subgroups showed moderate positive effects, and the effect size for short intervention periods of ≤3 weeks was slightly lower than that for the other duration groups. This preliminary observation is partially consistent with the conclusions of Li et al. (2026) in their review of exercise interventions for autistic individuals, namely that longer intervention periods are crucial for promoting more enduring and profound behavioral changes, whereas shorter periods tend to reflect short-term effects and immediate impacts (Li et al., 2026).

#### 5.3.3 Measurement tools and AI technology

Regarding measurement tools, the effect sizes for both behavioral observation and standardized scales were greater than 0.5 and statistically significant (*g* = 0.758, *g* = 0.546), further confirming the positive contribution of these measurement tools to intervention outcomes. Multiple studies have demonstrated that measurement tools influence research outcomes. For example, standardized measurement tools currently used to assess the final outcomes of autistic children lack representativeness and cannot accurately measure their level of improvement (Reichow and Wolery, 2009). Furthermore, significant methodological differences in outcome measurement hinder direct comparisons (Linstead et al., 2017). This explains the differences in effect sizes observed across the various measurement tools in this study, with the highest effect size observed when using “OBSERVE.” Such differences may stem from subjective biases inherent in behavioral observation itself. For example, participants may provide only positive information in self-reports due to misunderstandings, thereby generating so-called “good data” (DiTomasso and Colameco, 1982). Researchers also exhibit this psychological tendency. Particularly in empirical research, researchers often tend to report positive data that supports their design hypotheses in order to validate the effectiveness of their products, thereby reinforcing the theoretical frameworks or design concepts underpinning their work.

The bias introduced by this measurement tool may cause the pooled effect size to deviate from the true effect and lead practitioners to form biased or unrealistic expectations regarding the intervention’s effectiveness. Furthermore, theoretical models of the underlying intervention mechanisms may be unduly optimistic as a result, affecting the direction of research protocol optimization. Therefore, future empirical studies should prioritize the use of more objective and standardized assessment methods when selecting measurement tools to minimize the potential influence of assessor bias and enhance the reliability and external validity of research findings.

In the moderation analysis, AI technology was the only variable with a significant effect, indicating that different AI technologies or combinations thereof produce varying outcomes, which significantly increased the heterogeneity among studies. Specifically, adaptive learning technology (*g* = 0.780, *p* < 0.01) and computer vision technology combined with deep learning (*g* = 0.952, *p* < 0.001) achieved a significant large effect size. Machine learning (*g* = 0.412, *p* < 0.5) and standalone computer vision (*g* = 0.295, *p* < 0.01) demonstrated moderate to small effect sizes in their applications. In contrast, neurofeedback technology (*g* = 0.199, *p* > 0.05) exhibited a low and non-significant effect size, demonstrating limited therapeutic efficacy and inconsistent results for autistic children. These variations in effect sizes may stem from significant differences among AI technologies in terms of algorithmic complexity, computational demands, and maturity (Ide and Talamàs, 2025). Although AI technologies have found applications in the clinical treatment of autistic children (Elbattah et al., 2024). For example, convolutional neural networks can effectively recognize these children’s grasping movements (Zunino et al., 2018), and computer vision can provide real-time feedback on motor adjustments (de Belen et al., 2020).

However, the practical implementation of AI technology still faces numerous real-world challenges. These challenges primarily stem from limitations in signal quality. In particular, while neurofeedback technology can provide real-time monitoring and feedback for autistic individuals through electroencephalogram (EEG) or blood oxygen level signals (Alhwaiti, 2026), EEG signals are inherently fragile. Various sources of noise and interference in real-world environments can lead to signal sparsity, which directly compromises data quality (Zhang et al., 2025). More critically, the pediatric brain is in a rapid developmental phase, and prolonged use of brain-computer interface devices can cause fatigue and discomfort (Kinney-Lang et al., 2020), which in turn affects monitoring effectiveness. Some studies have directly pointed out that the clinical improvement effects of neurofeedback on neurodivergent children are not significant, with no notable changes observed in parent-reported scales or other cognitive indicators (Lam et al., 2022). The combination of these issues suggests that the reliability and effectiveness of current AI technology in real-world clinical settings still need to be improved.

### 5.4 Comprehensive evaluation of AI-driven gamified interventions

Although AI-driven gamified interventions offer unprecedented opportunities, their application also carries certain potential risks. These risks may not only undermine the overall effectiveness of the interventions but also cause unintended harm to the already vulnerable population of autistic children.

#### 5.4.1 Potential risks

In 2013, the American Psychiatric Association’s DSM-5 officially incorporated abnormal responses to sensory stimuli into the diagnostic criteria for autism spectrum disorder. Specifically, individuals may exhibit excessive or deficient responses to sensory input, or display unusual interests in environmental sensory aspects (American Psychiatric Association, 2013). Many individuals with autism are extremely sensitive to environmental stimuli such as sound, light, and touch. Such intense or unpredictable sensory input can easily trigger sensory overload (Brouche et al., 2024), which may lead to symptoms such as inattention, anger, and anxiety. In pursuit of “fun” and “immersion,” AI-driven games often incorporate various gamified elements, such as rapidly flashing visuals, multi-dimensional interactions, varying difficulty levels, and jarring sound effects. This increases difficulties for patients in selecting, organizing, and managing information (Taub et al., 2020), resulting in reduced responsiveness to the game and lower engagement (Costello and Donovan, 2019; Zappalà, 2023). This also requires us to further consider that more gamification elements are not necessarily better; the appropriate level of gamification remains to be determined.

The core objective of AI-driven games is to enhance the practical skills of autistic children, but the intervention medium typically involves the child’s solitary interaction with a screen or virtual characters, which inherently has non-social or even anti-social characteristics. This creates a profound paradox: we are attempting to teach social skills through a method that may reduce real-life interpersonal contact. A long-standing challenge regarding the generalization gap is how to ensure that skills practiced in SG are effectively transferred to real-life settings (Curcio et al., 2025). Such digital games allow interaction with others without the need for eye contact, but this can isolate children from reality, leading them to become dependent on the game and trapped in an idealized utopia. Furthermore, the current lack of standardized assessment and comparative frameworks for AI-enhanced game-based interventions limits the replicability of research and practical application (Kasari et al., 2021).

AI has long been a topic of controversy and debate, with the primary focus being on ethical and moral issues (Adams et al., 2022). While this ability to monitor AI behavior provides the technical foundation for personalized interventions, it also opens a “Pandora’s box” of ethical risks and data privacy concerns. Scholars such as Aastha Pant, focusing on the perspective of AI practitioners, found through surveys that only 3.38% of practitioners are familiar with all ethical principles. They highlight challenges facing AI systems, including a lack of shared understanding and foresight (Pant et al., 2024). AI-driven games are considered “intelligent” because they can retain data such as video recordings, therapy session records, and clinical records (Sohn et al., 2025). Consequently, establishing robust ethical oversight mechanisms and transparent data management strategies to safeguard the interests of autistic children has become imperative.

#### 5.4.2 Implementation barriers

Even if we can design AI games that are theoretically effective and safe, we still face a series of significant barriers in successfully scaling them up from laboratory settings or small-scale pilot studies to large-scale, diverse real-world environments. Recent research indicates that AI tools are viewed as technologies with significant potential. By dynamically adapting learning content, providing immediate feedback, using data analytics to predict student performance, and leveraging special features to enhance accessibility, AI tools effectively meet intervention requirements (Mitsea et al., 2025). However, the development process for AI-driven serious games is complex and costly, requiring robust technical infrastructure (Frutos-Pascual and Zapirain, 2015) and collaboration among multidisciplinary teams of experts (Marchiori et al., 2012). To ensure the effective adoption of new technologies, practitioners must undergo systematic training. Furthermore, the ongoing maintenance and updating of AI systems involve multifaceted resource requirements, including data privacy protection, software update deployment, and the assurance of reliable network connectivity; the resources and technical capabilities required for these tasks are often limited in resource-constrained environments (Sohn et al., 2025). This makes the development of a high-quality, evidence-based AI-driven therapeutic game an extremely costly investment.

### 5.5 Future game design recommendations

To address the emerging trend of gamified interventions in the treatment of autistic children, this study proposes a game design framework for future researchers or game designers, focusing on threer core game elements—feedback, rewards and Level Design—alongside AI application pathways. Future researchers or game designers must achieve high timeliness (feedback rate ≥ 80%) and accuracy in feedback delivery. They should employ real-time capture of children’s perceptual data through multimodal channels (visual, tactile, auditory) and provide diagnostic explanations during feedback, clearly identifying causes of errors or anomalies to facilitate behavioral correction. Reward design should incorporate a tiered structure, blending external incentives like points, badges, and virtual currency with intrinsic rewards such as self-efficacy and social recognition in specific proportions. This approach satisfies immediate motivation while cultivating long-term intrinsic motivation. Level design adheres to the principle of the Zone of Proximal Development, employing reinforcement learning for adaptive difficulty scaling. This ensures each level maintains a success rate between 60 and 80%, preventing excessive frustration or boredom. Transferring real-world context elements is also crucial.

Regarding AI implementation, three pathways should be prioritized: Data collection and annotation: Utilize cameras and wearable sensors to record children’s interactive behaviors and emotional physiological signals, enabling real-time feedback acquisition. Continuous algorithmic refinement: Drive iterative intervention loops through user feedback and behavioral analysis-based updates. Professional therapist supervision: Integrate expert oversight for efficient human-machine collaboration. Through the systematic integration of these strategies and pathways, precise and sustainable interventions for autistic children can be achieved while ensuring technological feasibility, providing a quantifiable and reproducible design paradigm for subsequent research.

### 5.6 Limitations

Although this study systematically reviews the current evidence on the effectiveness of AI-driven games for autistic children, this meta-analysis still has certain limitations. First, while this study collected empirical literature on AI-driven games for ASD intervention, the limited number of such studies designed for autistic children and the absence of corresponding data in most publications may compromise the generalizability of findings. Due to access constraints, only 14 studies with 37 effect sizes were ultimately included. This may indirectly affect the robustness of the findings. Second, the meta-analysis revealed significant heterogeneity among the included studies; therefore, the moderation analysis conducted in this study may not be exhaustive. Furthermore, given the limited body of evidence, this analysis is intended to be exploratory only. Therefore, future research could attempt to obtain relevant literature from additional sources to enrich the current body of evidence. Alternatively, further analysis could explore differences in intervention goal attainment between experimental and control groups of autistic children and delve deeper into the underlying causes.

## 6 Conclusion

This study employed a three-stage meta-analysis approach to systematically retrieve, screen, and comprehensively evaluate empirical research literature on “AI-driven game interventions for autistic children’s behavior” from major databases since their inception. It verified that no internal heterogeneity existed within the literature, with heterogeneity primarily stemming from differences between studies. Furthermore, moderation analyses explored how study design, measurement tools, AI technology, intervention frequency, and intervention objectives influenced outcomes. Results indicated that the complexity and maturity of underlying algorithms in different AI technologies were the primary drivers of heterogeneity across studies. Additionally, the study quantified the frequency of gamification elements, identifying three core components—feedback, rewards and levels—and described their key characteristics. Additionally, the study subdivided intervention objectives, revealing that AI-based games generally exert a positive influence on autistic children. Current interventions for this population primarily focus on emotional recognition and expression, demonstrating significant improvements in the combined development of “social + attention” skills. This demonstrates that AI-driven games can effectively promote multifaceted development in autistic children. Future research should consider incorporating AI technology to more efficiently identify children’s behaviors, dynamically adjust game designs for personalized, real-time feedback, and explore intervention targets such as attention, anxiety, and communication to enrich research outcomes.

## References

[ref1] Abd-alrazaq A. Abuelezz I. Hassan A. AlSammarraie A. Alhuwail D. Irshaidat S. . (2022). Artificial intelligence–driven serious games in health care: scoping review. JMIR Serious Games 10:e39840. doi: 10.2196/39840, 36445731 PMC9748798

[ref2] Adams C. Pente P. Lemermeyer G. Turville J. Rockwell G. (2022). Artificial intelligence and teachers’ new ethical obligations. Int. Rev. Inform. Ethics 31. doi: 10.29173/irie483

[ref3] Alhwaiti M. (2026). Application of neurofeedback in autism spectrum disorder: a systematic review and meta-analysis. Int. J. Dev. Disabil., 1–13. doi: 10.1080/20473869.2026.265140641624027

[ref4] Almurashi H. Bouaziz R. Alharthi W. Al-Sarem M. Hadwan M. Kammoun S. (2022). Augmented reality, serious games and picture exchange communication system for people with ASD: systematic literature review and future directions. Sensors 22:1250. doi: 10.3390/s22031250, 35161995 PMC8840490

[ref5] American Psychiatric Association (2013). Diagnostic and Statistical Manual of Mental Disorders. Fifth Edn Arlington, VA: American Psychiatric Publishing.

[ref6] Annunziata S. Santos L. Caglio A. Geminiani A. Brazzoli E. Piazza E. . (2024). Interactive mirroring games with social robot (IOGIOCO): a pilot study on the use of intransitive gestures in a sample of Italian preschool children with autism spectrum disorder. Front. Psych. 15:1356331. doi: 10.3389/fpsyt.2024.1356331, 39006819 PMC11240845

[ref7] Assink M. Wibbelink C. J. M. (2016). Fitting three-level meta-analytic models in R: a step-by-step tutorial. Quantitative Methods Psychol. 12, 154–174. doi: 10.20982/tqmp.12.3.p154

[ref8] Barmpakas A. Xinogalos S. (2023). Designing and evaluating a serious game for learning artificial intelligence algorithms: SpAI war as a case study. Appl. Sci. 13:5828. doi: 10.3390/app13105828

[ref11] Brouche S. Rigal N. Cazalis F. (2024). Parental descriptions of sensory processing in autism. Res. Autism Spectr. Disord. 118:102488. doi: 10.1016/j.rasd.2024.102488

[ref12] Carneiro T. Carvalho A. Frota S. Filipe M. G. (2024). Serious games for developing social skills in children and adolescents with autism Spectrum disorder: a systematic review. Healthcare 12:508. doi: 10.3390/healthcare12050508, 38470619 PMC10931397

[ref13] Casu M. Triscari S. Battiato S. Guarnera L. Caponnetto P. (2024). AI chatbots for mental health: a scoping review of effectiveness, feasibility, and applications. Appl. Sci. 14:5889. doi: 10.3390/app14135889

[ref14] Cheung M. W.-L. (2014). Modeling dependent effect sizes with three-level meta-analyses: a structural equation modeling approach. Psychol. Methods 19, 211–229. doi: 10.1037/a0032968, 23834422

[ref15] Costello R. Donovan J. (2019). How game designers can account for those with autism spectrum disorder (ASD) when designing game experiences. Int. J. End-User Comput. Dev. 8, 29–55. doi: 10.4018/IJEUCD.20190701.oa1

[ref16] Curcio E. Stasolla F. Zullo A. Di Gioia M. Passaro A. (2025). Enhancing socio-Emotional Skills in Children with Autism through AI-Powered Serious games: A Narrative Review. FedCSIS, 44, 15–21. doi: 10.15439/2025F7538

[ref17] de Belen R. A. J. Bednarz T. Arcot S. Del Favero D. (2020). Computer vision in autism spectrum disorder research: a systematic review of published studies from 2009 to 2019. Transl. Psychiatry 10:333. doi: 10.1038/s41398-020-01015-w, 32999273 PMC7528087

[ref18] Derks S. Willemen A. M. Sterkenburg P. S. (2022). Improving adaptive and cognitive skills of children with an intellectual disability and/or autism spectrum disorder: meta-analysis of randomised controlled trials on the effects of serious games. Int. J. Child-Comput. Interact. 33:100488. doi: 10.1016/j.ijcci.2022.100488

[ref19] DiTomasso R. A. Colameco S. (1982). Patient self-monitoring of behavior. J. Fam. Pract. 15, 79–83, 7086385

[ref9001] Duan Z. Wang X. Zhang Z. Wang X. Zhang Y. Du X. (2025). Digital and telehealth behavioral sleep interventions for improving sleep outcomes in children and adolescents with autism spectrum disorder: A systematic review and meta-analysis. Sleep Medicine 136:106870. doi: 10.1016/j.sleep.2025.106870, 41110404

[ref20] Elbattah M. Ibrahim O. A. S. Dequen G. (2024). Editorial: improving autism spectrum disorder diagnosis using machine learning techniques. Front. Neuroinform. 18:1529839. doi: 10.3389/fninf.2024.1529839, 39712346 PMC11659280

[ref21] Fakhrou A. Abdelazeem A. S. Hassanein E. E. A. (2024). The effects of AI-driven serious video games on facial expression abilities and academic performance of children with autism spectrum disorder: an empirical study. Lex Localis - J. Local Self-Gov. 22, 495–509. doi: 10.52152/s74hd313

[ref22] Fan M. Jin S. Fan J. Guo W. Chen X. (2025). EmoLand: utilizing narrative animations, multilevel games, and affective computing to foster emotional development in children with autism spectrum disorder. Int. J. Hum.-Comput. Stud. 199:103486. doi: 10.1016/j.ijhcs.2025.103486

[ref23] Fang Y.-M. Chen K.-M. Huang Y.-J. (2016). Emotional reactions of different interface formats: comparing digital and traditional board games. Adv. Mech. Eng. 8. doi: 10.1177/1687814016641902

[ref24] Faria D. R. da Silva Ayrosa P. P. (2025). Adaptive neuro-affective engagement via Bayesian feedback learning in serious games for neurodivergent children. Appl. Sci. 15:7532. doi: 10.3390/app15137532

[ref25] Ferrer R. Ali K. Hughes C. (2024). Using AI-based virtual companions to assist adolescents with autism in recognizing and addressing cyberbullying. Sensors 24:3875. doi: 10.3390/s24123875, 38931659 PMC11207624

[ref26] Freitas É. V. d. S. Panceri J. A. C. Schreider S. d. L. Caldeira E. M. d. O. Filho T. F. B. (2024). Cognitive serious games dynamically modulated as a therapeutic tool for applied behavior analysis therapy in children with autism spectrum disorder. Int. J. Emerging Technol. Learn. 19, 80–92. doi: 10.3991/ijet.v19i05.48677

[ref27] Frutos-Pascual M. Zapirain B. G. (2015). Review of the use of AI techniques in serious games: decision making and machine learning. IEEE Trans. Comput. Intell. AI Games 9, 133–152. doi: 10.1109/TCIAIG.2015.2512592

[ref28] Ganggayah M. D. Zhao D. Liew E. J. Y. Nor N. A. M. Paramasivam T. Lee Y. Y. . (2025). Accelerating autism spectrum disorder care: a rapid review of data science applications in diagnosis and intervention. Asian J. Psychiatr. 108:104498. doi: 10.1016/j.ajp.2025.104498, 40252472

[ref29] Gepner B. Féron F. (2009). Autism: a world changing too fast for a mis-wired brain? Neurosci. Biobehav. Rev. 33, 1227–1242. doi: 10.1016/j.neubiorev.2009.06.006, 19559043

[ref30] Ghiglino D. Floris F. De Tommaso D. Severino Russi N. Frulli A. Moretti S. . (2025). Enhancing theory of mind in autism through humanoid robot interaction in a randomized controlled trial. Sci. Rep. 15:27650. doi: 10.1038/s41598-025-12253-7, 40731038 PMC12307709

[ref31] Govender T. Arnedo-Moreno J. (2021). An analysis of game design elements used in digital game-based language learning. Sustainability 13:6679. doi: 10.3390/su13126679

[ref32] Hallifax S. Lavoué E. Serna A. (2020). To tailor or not to tailor gamification? An analysis of the impact of tailored game elements on learners’ behaviours and motivation. Lect. Notes Comput. Sci 12163, 216–227. doi: 10.1007/978-3-030-52237-7_18, 42287054

[ref33] Hansen S. G. Mowbray M. Raulston T. Carnett A. Tullis C. (2023). Effects of a peer-mediated joint attention intervention in an inclusive preschool setting. Focus Autism Dev. Disabil. 38, 71–79. doi: 10.1177/10883576221108111

[ref34] Hassan A. Pinkwart N. Shafi M. (2025). Zirkus Empathico 2.0: a multiplayer serious mobile game for children with autism spectrum disorder (ASD), with a focus on enhancing social and emotional development. Multimed. Tools Appl. 84, 41947–41970. doi: 10.1007/s11042-025-20826-x

[ref35] Higgins J. P. Thompson S. G. Deeks J. J. Altman D. G. (2003). Measuringinconsistency in meta-analyses. Br. Med. J. 327, 557–560. doi: 10.1136/bmj.327.7414.557, 12958120 PMC192859

[ref36] Hong Y. Saab N. Admiraal W. (2024). Approaches and game elements used to tailor digital gamification for learning: a systematic literature review. Comput. Educ. 212:105000. doi: 10.1016/j.compedu.2024.105000

[ref37] Huber S. E. Cortez R. Kiili K. Lindstedt A. Ninaus M. (2023). Game elements enhance engagement and mitigate attrition in online learning tasks. Comput. Hum. Behav. 149:107948. doi: 10.1016/j.chb.2023.107948

[ref38] Iannone A. Giansanti D. (2024). Breaking barriers—the intersection of AI and assistive Technology in Autism Care: a narrative review. J. Personalized Med. 14:41. doi: 10.3390/jpm14010041, 38248742 PMC10817661

[ref39] Ide E. Talamàs E. (2025). Artificial intelligence in the knowledge economy. J. Polit. Econ. 133, 3762–3800. doi: 10.1086/737233

[ref40] Ji B. Batubara I. M. S. Batten J. Peng X. Chen S. Ni Z. (2025). Digital health interventions targeting psychological health in parents of children with autism spectrum disorder: protocol for a scoping review. JMIR Res. Protocols 14:e68677. doi: 10.2196/68677, 40466097 PMC12177426

[ref41] Jouen A. L. Narzisi A. Xavier J. Tilmont E. Bodeau N. Bono V. . (2017). GOLIAH (gaming open library for intervention in autism at home): a 6-month single blind matched controlled exploratory study. Child Adolesc. Psychiatry Ment. Health 11:17. doi: 10.1186/s13034-017-0154-7, 28344643 PMC5361849

[ref42] Karami B. Koushki R. Arabgol F. Rahmani M. Vahabie A.-H. (2021). Effectiveness of virtual/augmented reality–based therapeutic interventions on individuals with autism spectrum disorder: a comprehensive meta-analysis. Front. Psych. 12:665326. doi: 10.3389/fpsyt.2021.665326, 34248702 PMC8260941

[ref43] Kasari C. Shire S. Shih W. Almirall D. (2021). Getting SMART about social skills interventions for students with ASD in inclusive classrooms. Except. Child. 88, 26–44. doi: 10.1177/00144029211007148

[ref44] Kent C. Cordier R. Joosten A. Wilkes-Gillan S. Bundy A. Speyer R. (2020). A systematic review and meta-analysis of interventions to improve play skills in children with autism spectrum disorder. Rev. J. Autism Dev. Disord. 7, 91–118. doi: 10.1007/s40489-019-00181-y

[ref45] Khatab S. Hijab M. H. F. Othman A. Al-Thani D. (2024). Collaborative play for autistic children: a systematic literature review. Entertainment Comp. 50:100653. doi: 10.1016/j.entcom.2024.100653

[ref46] Kinkel S. Baumgartner M. Cherubini E. (2022). Prerequisites for the adoption of AI technologies in manufacturing – evidence from a worldwide sample of manufacturing companies. Technovation 110:102375. doi: 10.1016/j.technovation.2021.102375

[ref47] Kinney-Lang E. Kelly D. Floreani E. D. Jadavji Z. Rowley D. Zewdie E. T. . (2020). Advancing brain-computer interface applications for severely disabled children through a multidisciplinary national network: summary of the inaugural pediatric BCI Canada meeting. Front. Hum. Neurosci. 14:593883. doi: 10.3389/fnhum.2020.593883, 33343318 PMC7744376

[ref48] Kouroupa A. Laws K. R. Irvine K. Mengoni S. E. Baird A. Sharma S. (2022). The use of social robots with children and young people on the autism spectrum: a systematic review and meta-analysis. PLoS One 17:e0269800. doi: 10.1371/journal.pone.0269800, 35731805 PMC9216612

[ref49] Kuo M. H. Orsmond G. I. Cohn E. S. Coster W. J. (2013). Friendship characteristics and activity patterns of adolescents with an autism spectrum disorder. Autism 17, 481–500. doi: 10.1177/1362361311416380, 22087043

[ref50] Lam S.-L. Criaud M. Lukito S. Westwood S. J. Agbedjro D. Kowalczyk O. S. . (2022). Double-blind, sham-controlled randomized trial testing the efficacy of fMRI neurofeedback on clinical and cognitive measures in children with ADHD. Am. J. Psychiatry 179, 947–958. doi: 10.1176/appi.ajp.21100999, 36349428 PMC7614456

[ref51] Li G. Mohammad A. Z. Alibakhshi G. Labbafi A. (2024). Teachers and educators’ experiences and perceptions of artificial-powered interventions for autism groups. BMC Psychol. 12, 199–112. doi: 10.1186/s40359-024-01664-2, 38605422 PMC11010416

[ref52] Li Y. Qi Y. Yang Y. Zhang R. (2026). Effects of exercise intervention on inhibitory control in children and adolescents with autism spectrum disorders: a systematic review and meta-analysis. Medicine 105:e47186. doi: 10.1097/MD.0000000000047186, 41560031 PMC12826300

[ref53] Li J. Zheng Z. Chai Y. Li X. Wei X. (2023). Faceme: an agent-based social game using augmented reality for the emotional development of children with autism spectrum disorder. Int. J. Hum.-Comput. Stud. 175:103032. doi: 10.1016/j.ijhcs.2023.103032

[ref55] Linstead E. Dixon D. R. French R. Granpeesheh D. Adams H. German R. . (2017). Intensity and learning outcomes in the treatment of children with autism spectrum disorder. Behav. Modif. 41, 229–252. doi: 10.1177/0145445516667059, 27651097

[ref56] López-Bouzas N. del Moral-Pérez M. E. Castañeda-Fernández J. (2024). Improved socio-emotional skills in students with autism spectrum disorder (ASD) following an intervention supported by an augmented gamified environment. Int. J. Child-Comput. Interact. 42:100683. doi: 10.1016/j.ijcci.2024.100683

[ref57] Lorusso D. Melchiorre L. Toto G. A. (2025). The use of Educational Robotics in Autism, vol. 2521 ICS exchange, 373–386. Cham, Switzerland: Springer.

[ref58] Luongo M. Simeoli R. Marocco D. Ponticorvo M. (2022) The design of a game-based software for children with autism spectrum disorder. In 2022 IEEE International Conference on Metrology for Extended Reality, Artificial Intelligence and Neural Engineering, 318–322.

[ref59] Lyu Y. Liu D. An P. Tong X. Zhang H. Katsuragawa K. . (2024). Emooly: supporting autistic children in collaborative social-emotional learning with caregiver participation through interactive AI-infused and AR activities. Proc. ACM Interact. Mob. Wearable Ubiquitous Technol. 8, 1–36. doi: 10.1145/3699738

[ref60] Marchiori E. J. Torrente J. del Blanco Á. Moreno-Ger P. Sancho P. Fernández-Manjón B. (2012). A narrative metaphor to facilitate educational game authoring. Comput. Educ. 58, 590–599. doi: 10.1016/j.compedu.2011.09.017

[ref61] Martins S. R. A. L. Souza F. A. Teixeira L. C. Leal B. R. A. Silva D. M. P. F. Silva C. M. R. (2026). Towards screening of children students with autism spectrum disorder based on executive functions with serious game and machine learning approaches. Expert Syst. Appl. 295:128884. doi: 10.1016/j.eswa.2025.128884

[ref62] Mayer R. E. (2005). Cognitive theory of multimedia learning. The Cambridge handbook of multimedia learning, 41, 31–48. doi: 10.1017/CBO9780511816819.004

[ref63] Mazarakis A. Bräuer P. (2023). Gamification is working, but which one exactly? Results from an experiment with four game design elements. Int. J. Hum.-Comput. Interact. 39, 612–627. doi: 10.1080/10447318.2022.2041909

[ref64] Mehmood F. Mahzoon H. Yoshikawa Y. Ishiguro H. Sadia H. Ali S. (2021). Attentional behavior of children with ASD in response to robotic agents. IEEE Access 9, 31946–31955. doi: 10.1109/ACCESS.2021.3056211

[ref65] Mir H. Y. Khosla A. K. (2018) Kinect based game for improvement of sensory, motor and learning skills in autistic children Proceedings of 2018 Second International Conference on Intelligent Computing and Control Systems (pp. 1670–1674)

[ref66] Mitsea E. Drigas A. Skianis C. (2025). A systematic review of serious games in the era of artificial intelligence, immersive technologies, the metaverse, and neurotechnologies: transformation through meta-skills training. Electronics 14:649. doi: 10.3390/electronics14040649

[ref67] Mittal P. Bhadania M. Tondak N. Ajmera P. Yadav S. Kukreti A. . (2024). Effect of immersive virtual reality-based training on cognitive, social, and emotional skills in children and adolescents with autism spectrum disorder: a meta-analysis of randomized controlled trials. Res. Dev. Disabil. 151:104771. doi: 10.1016/j.ridd.2024.104771, 38941690

[ref68] Muhammad S. J. Rizwan A. K. (2019). Can autism be catered with artificial intelligence-assisted intervention technology? A comprehensive survey. Artif. Intell. Rev. 53, 1039–1069. doi: 10.1007/s10462-019-09686-8, 30311153

[ref69] Nakagawa S. Yang Y. Macartney E. L. Spake R. Lagisz M. (2023). Quantitative evidence synthesis: a practical guide on meta-analysis, meta-regression, and publication bias tests for environmental sciences. Environ. Evid. 12:8. doi: 10.1186/s13750-023-00301-6, 39294795 PMC11378872

[ref70] O’Keeffe C. McNally S. (2023). A systematic review of play-based interventions targeting the social communication skills of children with autism spectrum disorder in educational contexts. Rev. J. Autism Dev. Disord. 10, 51–81. doi: 10.1007/s40489-021-00286-3

[ref71] Paengkumhag C. Limpornchitwilai W. Supaluk S. Chamnongthai K. Kaewkamnerdpong B. (2025). Enhancing ADL skill acquisition in children with ASD through a personalized, fuzzy logic-based tablet game: a pilot study. Sci. Rep. 15:37691. doi: 10.1038/s41598-025-21586-2, 41152409 PMC12568980

[ref72] Pandya S. Jain S. Verma J. (2024). A comprehensive analysis towards exploring the promises of AI-related approaches in autism research. Comput. Biol. Med. 168:107801. doi: 10.1016/j.compbiomed.2023.107801, 38064848

[ref73] Pant A. Hoda R. Spiegler S. V. Tantithamthavorn C. Turhan B. (2024). Ethics in the age of AI: an analysis of AI practitioners’ awareness and challenges. ACM Trans. Softw. Eng. Methodol. 33, 1–35. doi: 10.1145/3635715

[ref74] Penev Y. Dunlap K. Husic A. Hou C. Washington P. Leblanc E. . (2021). A mobile game platform for improving social communication in children with autism: a feasibility study. Appl. Clin. Inform. 12, 1030–1040. doi: 10.1055/s-0041-1736626, 34788890 PMC8598393

[ref75] Pigott T. D. Polanin J. R. (2020). Methodological guidance paper: high-quality meta-analysis in a systematic review. Rev. Educ. Res. 90, 24–46. doi: 10.3102/0034654319877153

[ref76] Priyadharshan R. Narendhar N. Menon M. V. Gayathri M. Kumar D. A. Ganapathy Sankar U. (2025) Design and development of console-based gaming for balance training in autism spectrum disorder children 2025 2nd International Conference on Trends in Engineering Systems and Technologies (pp. 1–5).

[ref77] Reichow B. Wolery M. (2009). Comprehensive synthesis of early intensive behavioral interventions for young children with autism based on the UCLA young autism project model. J. Autism Dev. Disord. 39, 23–41. doi: 10.1007/s10803-008-0596-0, 18535894

[ref78] Rothstein H. Sutton A. J. Borenstein M. (2005). Publication bias in meta-Analysis: Prevention, Assessment, and Adjustments. Hoboken, NJ: Wiley.

[ref79] Schreibelmayr S. Mara M. (2025). Determinants of self-reported and behavioral trust in an AI advisor within a cooperative problem-solving game. Comp. Human Behav. Artificial Humans 6:100235. doi: 10.1016/j.chbah.2025.100235, 38826717

[ref80] Shim S. R. Kim S. J. Lee J. Rücker G. (2019). Network meta-analysis: application and practice using R software. Epidemiol. Health 41:e2019013. doi: 10.4178/epih.e2019013, 30999733 PMC6635665

[ref81] Shohieb S. M. Doenyas C. Elhady A. M. (2022). Dynamic difficulty adjustment technique-based mobile vocabulary learning game for children with autism spectrum disorder. Entertain. Comput. 42:100495. doi: 10.1016/j.entcom.2022.100495

[ref82] Sierra I. Díaz-Díaz N. Barranco C. Carrasco-Villalón R. (2022). Artificial intelligence-assisted diagnosis for early intervention patients. Appl. Sci. 12:8953. doi: 10.3390/app12188953

[ref83] Silva M. R. d. V. C. e. Gaigg S. B. Benjamin L. Bogosian A. (2025). Interventions to improve parental mental health and psychological well-being in parents of adolescents with a diagnosis of ASD and/or ADHD: a systematic review. Res. Autism 126:202649. doi: 10.1016/j.reia.2025.202649

[ref84] Sohn J.-S. Lee E. Kim J.-J. Oh H.-K. Kim E. (2025). Implementation of generative AI for the assessment and treatment of autism spectrum disorders: a scoping review. Front. Psych. 16:1628216. doi: 10.3389/fpsyt.2025.1628216, 40766925 PMC12322814

[ref85] Soler-Bages M. (2025) Enhancing accessibility in XR games for users with autism spectrum disorder. In 2025 IEEE Conference on Virtual Reality and 3D User Interfaces (pp. 1572–1573)

[ref86] Sosnowski D. W. Stough C. O. Weiss M. J. Cessna T. Casale A. Foran A. . (2022). Brief report: a novel digital therapeutic that combines applied behavior analysis with gaze-contingent eye tracking to improve emotion recognition in children with autism spectrum disorder. J. Autism Dev. Disord. 52, 2357–2366. doi: 10.1007/s10803-021-05101-w, 34060003

[ref87] Sterne J. A. C. Hernán M. A. Reeves B. C. Savović J. Berkman N. D. Viswanathan M. . (2016). ROBINS-I: a tool for assessing risk of bias in non-randomised studies of interventions. BMJ 355:i4919. doi: 10.1136/bmj.i4919, 27733354 PMC5062054

[ref88] Sterne J. A. C. Savović J. Page M. J. Elbers R. G. Blencowe N. S. Boutron I. . (2019). RoB 2: a revised tool for assessing risk of bias in randomised trials. BMJ 366:l4898. doi: 10.1136/bmj.l4898, 31462531

[ref89] Taub M. Sawyer R. Smith A. Rowe J. Azevedo R. Lester J. (2020). The agency effect: the impact of student agency on learning, emotions, and problem-solving behaviors in a game-based learning environment. Comput. Educ. 147:103781. doi: 10.1016/j.compedu.2019.103781

[ref90] Todd J. T. Bahrick L. E. (2023). Individual differences in multisensory attention skills in autistic children predict language and symptom severity: evidence from the multisensory attention assessment protocol (MAAP). J. Autism Dev. Disord. 53, 4685–4710. doi: 10.1007/s10803-022-05752-3, 36181648 PMC10065966

[ref91] Toma M. V. Turcu C. E. Turcu C. O. Vlad S. Tiliute D. E. Pascu P. (2024). Extended reality–based mobile app solutions for the therapy of children with autism spectrum disorders: systematic literature review. JMIR Serious Games 12:e49906. doi: 10.2196/49906, 38373032 PMC10913001

[ref92] Tsikinas S. Xinogalos S. (2019). Studying the effects of computer serious games on people with intellectual disabilities or autism spectrum disorder: a systematic literature review. J. Comput. Assist. Learn. 35, 61–73. doi: 10.1111/jcal.12311

[ref93] Tupou J. (2020). Meta-analysis supports naturalistic developmental behavioral interventions as a promising approach for improving a range of outcomes for children with autism spectrum disorder. Evid. Based Commun. Assess. Interv. 14, 206–210. doi: 10.1080/17489539.2020.1816328

[ref94] Vacca R. A. Augello A. Gallo L. Caggianese G. Malizia V. La Grutta S. . (2023). Serious games in the new era of digital-health interventions: a narrative review of their therapeutic applications to manage neurobehavior in neurodevelopmental disorders. Neurosci. Biobehav. Rev. 149:105156. doi: 10.1016/j.neubiorev.2023.105156 37019246

[ref95] Valente S. M. (2004). Autism. J. Am. Psychiatr. Nurses Assoc. 10, 236–243. doi: 10.1177/1078390304269789

[ref96] Vukićević S. Đordević M. Glumbić N. Bogdanović Z. Đurić Jović M. (2019). A demonstration project for the utility of Kinect-based educational games to benefit motor skills of children with ASD. Percept. Mot. Skills 126, 1117–1144. doi: 10.1177/0031512519867521, 31390305

[ref97] Wang Z. Dou Y. Yang X. Guo X. Ma X. Zhou B. . (2025c). Global, regional, and national burden of mental disorders among adolescents and young adults, 1990–2021: a systematic analysis for the global burden of disease study 2021. Transl. Psychiatry 15:397. doi: 10.1038/s41398-025-03623-w, 41073427 PMC12514266

[ref98] Wang Q. Jia S. Cai Z. Jiang W. Wang X. Wang J. (2025a). The canonical correlation between executive function and social skills in children with autism spectrum disorder and potential pathways to physical fitness. Sci. Rep. 15:10367. doi: 10.1038/s41598-025-94334-1, 40133491 PMC11937234

[ref99] Wang T. Ma H. Ge H. Sun Y. Kwok T.-O. Liu X. . (2025b). The use of gamified interventions to enhance social interaction and communication among people with autism spectrum disorder: a systematic review and meta-analysis. Int. J. Nurs. Stud. 165:105037. doi: 10.1016/j.ijnurstu.2025.105037 40043470

[ref100] Wang K. Qiu F. Liu J. Yang X. (2026). The effects of exercise intervention for restricted and repetitive behavior in children with autism spectrum disorder: a network meta-analysis. J. Behav. Cognitive Therapy 36:100549. doi: 10.1016/j.jbct.2025.100549

[ref101] Wankhede N. Kale M. Shukla M. Nathiya D. Roopashree R. Kaur P. . (2024). Leveraging AI for the diagnosis and treatment of autism spectrum disorder: current trends and future prospects. Asian J. Psychiatr. 101:104241. doi: 10.1016/j.ajp.2024.104241, 39276483

[ref102] Weidlich J. Gašević D. Drachsler H. Kirschner P. (2025). ChatGPT in education: an effect in search of a cause. J. Comput. Assist. Learn. 41:e70105. doi: 10.1111/jcal.70105

[ref103] Wijnhoven L. A. M. W. Creemers D. H. M. Vermulst A. A. Lindauer R. J. L. Otten R. Engels R. C. M. E. . (2020). Effects of the video game ‘Mindlight’ on anxiety of children with an autism spectrum disorder: a randomized controlled trial. J. Behav. Ther. Exp. Psychiatry 68:101548. doi: 10.1016/j.jbtep.2020.101548, 32155470

[ref104] Wu Y. Yi A. Ma C. Chen L. (2023). Artificial intelligence for video-game visualization, advancements, benefits and challenges. Math. Biosci. Eng. 20, 15345–15373. doi: 10.3934/mbe.2023686, 37679183

[ref105] Xing X. Zhang Z. He W. (2026). AI technology, AI narrative, and firm value. Technovation 149:103349. doi: 10.1016/j.technovation.2025.103349

[ref106] Yang J. Li R. (2025). Systematic review and randomized controlled trial meta-analysis of the effects of physical activity interventions and their components on repetitive stereotyped behaviors in patients with autism spectrum disorder. Front. Psychol. 16:1579345. doi: 10.3389/fpsyg.2025.1579345, 40486887 PMC12141328

[ref107] Yang X. Wu J. Ma Y. Yu J. Cao H. Zeng A. . (2025). Effectiveness of virtual reality technology interventions in improving the social skills of children and adolescents with autism: systematic review. J. Med. Internet Res. 27:e60845. doi: 10.2196/60845, 39907288 PMC11840372

[ref109] Zappalà E. (2023). When the gaming gets serious: Reflections on student agency within inclusive virtual learning environments. Journal of Inclusive Methodology and Technology in Learning and Teaching. 3:55. doi: 10.32043/jimtlt.v3i1.55

[ref110] Zhang T. Zhou X. Li X. Wang Y. Fan Q. Liang J. . (2025). Noninvasive brain–computer interfaces for children with neurodevelopmental disorders: attention deficit hyperactivity disorder and autism spectrum disorder. Displays 86:102886. doi: 10.1016/j.displa.2024.102886

[ref111] Zhao K. Fan F. (2025). Educational interventions for autism spectrum disorder: past, present, and future perspectives. Int. J. Innov. Res. Med. Sci. 10, 407–413. doi: 10.23958/ijirms/vol10-i12/2145

[ref112] Zhou Q. Li D. Zhang Y. Zhang Q. Li Y. Zhu C. . (2025). Evaluating the effectiveness of intelligent interaction technology in autism interventions: a meta-analysis based on trial assessment. Res. Dev. Disabil. 164:105087. doi: 10.1016/j.ridd.2025.105087, 40784194

[ref113] Zunino A. Morerio P. Cavallo A. Ansuini C. Podda J. Battaglia F. (2018) Video gesture analysis for autism spectrum disorder detection Proceedings of the 24th International Conference on Pattern Recognition (ICPR) (pp. 3421–3426)

[ref114] Zuo R. Li W. Zhang X. (2025). The impact of information and communication technology (ICT) on learning outcomes in early childhood and primary education: a meta-analysis of moderating factors. Front. Psychol. 16:1540169. doi: 10.3389/fpsyg.2025.1540169, 40606877 PMC12213516

